# P68 - Immunogenicity of 13-valent conjugate pneumococcal vaccine (PCV13) in children with asthma or recurrent wheeze receiving inhaled corticosteroids (ICS)

**DOI:** 10.1186/2045-7022-4-S1-P123

**Published:** 2014-02-28

**Authors:** Lefki Giannopoulou, Polytimi Panaghiotopoulou-Gartagani, Eleni Kyritsi, Athanasios Kaditis, Maria Theodoridou, Vasiliki Spoulou

**Affiliations:** 1 Pediatrics Department, Tzaneio General Hospital, Piraeus, Greece; 2 Pediatric Pulmonology Unit, 1st Department of Pediatrics, National Kapodistrian University of Athens School of Medicine, “Aghia Sophia” Children's Hospital, Athens, Greece; 3 Technological Institution of Athens, 1st Nursing Department, Athens, Greece; 4 1st Department of Pediatrics, National and Kapodistrian University of Athens School of Medicine, “Aghia Sophia” Children's Hospital, Athens, Greece

## Introduction

Patients with asthma have been reported to be at increased risk of invasive pneumococcal disease (IPD). Therefore, routine vaccination against Streptococcus Pneumoniae is suggested for all children suffering from asthma or recurrent wheeze. Inhaled corticosteroids (ICS) are the preferred treatment for initiating long-term control therapy in these children. Their effect on the immunogenicity of PCV13 is yet unknown.

## Aim

To evaluate the immunogenicity of PCV13, in children with asthma or recurrent wheeze, receiving medium doses of ICS for extended periods of time.

## Method

40 children (26 boys) with asthma or recurrent wheeze (mean age 4,3 ± 1,05 years) were assigned to 3 groups according to the duration of their ICS therapy (1st <30days, 2nd 30-90 days, 3rd >90 days, dosage 200-250μg/day of inhaled fluticasone in all groups). 13 healthy age-matched children were included in the study as controls (group 0). All children received for the first time one dose of PCV13. Pneumococcal serotype (PS) specific IgG antibodies to serotypes 3 and 19A were measured by ELISA, before and 31,9 ± 5,6 days after vaccination. IgG concentration ≥0,35μg/ml was considered protective for invasive disease. For the statistical analysis SPSS17 and non-parametric tests were used. The statistical significance level was set at p<0,05.

## Results

One month after vaccination, 96,2% of all 53 children had protective IgG concentration ≥0,35μg/ml, for each serotype. The geometric mean concentration (GMC) of IgG antibodies after vaccination, the increase in the GMC and the percentage of children that achieved protective IgG concentration ≥0,35μg/ml, did not differ for neither of the serotypes, when compared among our 4 groups (p > 0,05).

## Conclusion

The immunogenicity of PCV13, in children with asthma or recurrent wheeze, is not affected by the use of medium doses of ICS as control therapy, even after extended periods of time.

